# Effects of Oral Carotenoids on Oxidative Stress: A Systematic Review and Meta-Analysis of Studies in the Recent 20 Years

**DOI:** 10.3389/fnut.2022.754707

**Published:** 2022-04-27

**Authors:** Chengfei Zhuang, Jinping Yuan, Yimei Du, Jing Zeng, Yan Sun, Yan Wu, Xing-Hua Gao, Hong-Duo Chen

**Affiliations:** ^1^Department of Dermatology, The First Hospital of China Medical University, Shenyang, China; ^2^Department of Dermatology, The Second Affiliated Hospital of Harbin Medical University, Harbin, China

**Keywords:** carotenoids, oxidative stress, antioxidants, lipid/lipoprotein, age-related disease

## Abstract

Carotenoids protect organs, tissues, and cells from the damaging action of singlet oxygen, oxygen radicals, and lipid peroxides. This systematic review was sought to evaluate the influence of oral carotenoids on antioxidant/oxidative markers, blood carotenoids levels, and lipid/lipoprotein parameters in human subjects. A comprehensive review of relevant literature was conducted in PubMed, Web of Sciences, and the Cochrane library, from 2000 to December 2020. Randomized controlled trials, case-controlled trials, or controlled trials were identified. A total of eighteen trials were included, with the target populations being healthy subjects in 16 studies, athletes in 1 study, and pregnant women in 1 study. The meta-analysis results showed that carotenoids complex supplementation significantly increased the levels of antioxidative parameters ferric-reducing ability of plasma (FRAP) and oxygen radical absorbance capacity (ORAC) [standardized mean difference (SMD) = 0.468; 95% CI: 0.159–0.776, *p* = 0.003; SMD = 0.568; 95% CI: 0.190–0.947, *p* = 0.003] and decreased the blood triglyceride (TG) level (SMD = −0.410, 95% CI: −0.698 to −0.122, *p* = 0.005). Oral carotenoids supplement significantly increased the blood levels of β-carotene (SMD = 0.490, 95% CI: 0.123–0.858, *p* = 0.009), α-tocopherol (SMD = 0.752, 95%CI: 0.020–1.485, *p* = 0.044), and the intaking durations were 8 weeks. The levels of antioxidative enzymes and other lipid/lipoprotein parameters were not different between subjects receiving carotenoids and controls (*p* > 0.05). In conclusion, our systematic review showed that the carotenoids complex is beneficial for alleviating potential oxidative stress *via* interacting with free radicals or decreasing blood TG levels. The intaking duration of carotenoids should be 8 weeks to reach enough concentration for function.

## Introduction

Oxidative stress has been implicated in the etiology of several chronic diseases, namely, cardiovascular disease (1), type 2 diabetes (2), neurodegenerative disease (3), some cancers (4–6), and also involved in the aging process and age-related diseases (ARDs) (7). Aging is an unavoidable biological phenomenon affecting all multicellular organisms (8). Various hypotheses have been put forward to explain the molecular reasons for aging (9, 10). In damage theories, reactive oxygen species (ROS) is considered to lead to cumulative DNA, protein, and lipid damages, which play a prominent role in the pathogenesis and progression of aging (11).

Accumulation of ROS leads to inflammation, cellular dysfunction and cell death, and mitochondrial dysfunction. Decline in mitochondrial function, the oxidative stress response in aging, and accumulation of aberrant proteins may contribute to ARD (12). What is more, a specific form of oxidative stress called photo-oxidative stress (13) which is induced by UV exposure can cause a common external aging, i.e., photoaging (14). Uncontrolled production of ROS is implicated in vascular injury and oxidative stress participates in antioxidant mechanisms in the development and progression of atherosclerosis (15). ROS can also promote tumor formation by inducing DNA mutations and pro-oncogenic signaling pathways; oxidative stress is an important factor in both the tumor development and responses to anticancer therapies (16).

In this regard, carotenoids are of particular interest (12). Carotenoids act as electron-transport agents and play crucial roles in protecting organs, tissues, and cells from the damaging action of singlet oxygen, oxygen radicals, and lipid peroxides (17). Experimental studies have demonstrated that they reduce chemical-induced neoplasia (18), improve erythrocyte antioxidant status (19), and protect tissues from UV-related damage (20). According to chemical structure, carotenoids were generally classified as pure hydrocarbon carotenoids called “carotenes” (such as lycopene, α-carotene, β-carotene and β-cryptoxanthin) and carotenoids containing one or several oxygen functions known as “xanthophylls” (such as lutein, zeaxanthin) (21). Some precursors during biosynthesis such as lycopene also belong to carotenoids (21). Nowadays, carotenoids are the most numerous and widespread group of hydrophobic pigments mainly in fruits and vegetables (22). High fruit and vegetable consumption is linked with changes in specific antioxidant markers or early-disease indicators, for example, cholesterol oxidation products, plasma antioxidant capacity, oxidized DNA base damage, etc. (23–28). It has recently been hypothesized that carotenoids cannot be biosynthesized by humans and animals *de novo*, but can be derived from their food and feed, respectively (21).

To the best of our knowledge, the systematic investigation of the relationship between diary carotenoids and redox markers or lipid/lipoprotein parameters was lacking. The current systematic review was conducted to evaluate the influence of diary carotenoids on antioxidant/oxidative stress, blood carotenoids levels, and lipid/lipoprotein parameters, in human subjects. The study will add valuable evidence of carotenoids on improving disease caused by oxidative stress.

## Materials and Methods

### Search Strategy

A systematic review of the literature was conducted in the following databases: PubMed, Web of Sciences, the Cochrane library, and CNKI from 2000 to December 2020. The searching strategies were interventions (“antioxidant” or “carotenoid” or “carotene” or “lutein” or “lycopene” or “zeaxanthin” or “tocopherol” or “cryptoxanthin” or “canthaxanthin”), outcomes [“oxidative stress” or “ferric-reducing ability of plasma (FRAP)” or “oxygen radical absorbance capacity (ORAC)” or “superoxide dismutase (SOD)” or “catalase (CAT)”or “glutathione peroxidase (GPx)” or “lipid” or “lipoprotein” or “high density lipoprotein (HDL)” or “triglyceride (TG)”], and study designs (“random” or “control”). In addition, a hand-searching of the citation lists and the articles of the relevant publications were performed to identify other potentially eligible studies.

### Study Selection

The clinical trials with the following criteria were included in this systematic review: (1) randomized controlled trials (RCTs), case-controlled trials, and controlled trials; (2) human subjects being the target population without age limitation; (3) studies relating to oxidative stress, lipid/lipid-protein, or carotenoids level; and (4) treatment group receiving oral carotenoids supplementation (single carotenoid, carotenoids complex, or dietary botanical carotenoids) and control group (placebo, no treatment, or other treatment). Studies that lack necessary information were excluded.

### Data Extraction and Quality Assessment

Characteristics of eligible studies were extracted using a predesigned collection form. The data extracted included: The first author's name, publication year, country, subject numbers, gender, age, body mass index (BMI), weight, intervention, and outcome.

The methodological quality of each included study was assessed using the “the Cochrane Collaboration Risk of bias tool.” The assessment covers the following biases: random sequence generation (selection bias), allocation concealment (selection bias), blinding of participants and personnel (performance bias), blinding of outcome assessment (detection bias), incomplete outcome data (attrition bias), selective reporting (reporting bias), and other bias.

Two independent authors (Chengfei Zhuang and Yan Sun) did the above tasks of literature searching, study selection, data extraction, and risk of bias assessment. Upon any disagreement, a third author (Yan Wu) was resorted to reach a consensus.

### Statistical Analysis

All the statistical analyses were performed using STATA 13.0 software (Stata Corporation, College Station, Texas, USA). Moreover, RevMan V.5.3 software (Cochrane Collaboration, Oxford, UK) was used for risk of bias assessment. Standardized mean difference (SMD) with 95% CI was used to express the comparison results of two groups, based on the inverse variance method and Cohen statistics. When an outcome was measured in 2 or more studies, the pooled estimate was made. When outcomes were measured at 2 or more time points in the included studies, only data of baseline and final time point were extracted and the change levels of treatment and control group were compared. Subanalysis by supplements and time points was undertaken if there were enough studies to conduct a separate meta-analysis. The data were considered the significant difference between treatment and control groups when the *p* < 0.05. The results of the meta-analysis were presented as forest plots. Heterogeneity was determined using *p-*value and *I*^2^ statistics. The *I*^2^ > 50% with a *p* < 0.05 was denoted to be significant heterogeneity between the studies, and a random-effects model was adopted, or else, a fixed-effects model would be used. If obvious clinical heterogeneity existed, though statistical heterogeneity was not detected, a random effects model would be adopted. Publication bias was scrutinized using Egger's test with a *p* < 0.1 being representative of significant publication bias.

## Results

### Literature Search

**Figure 1:**
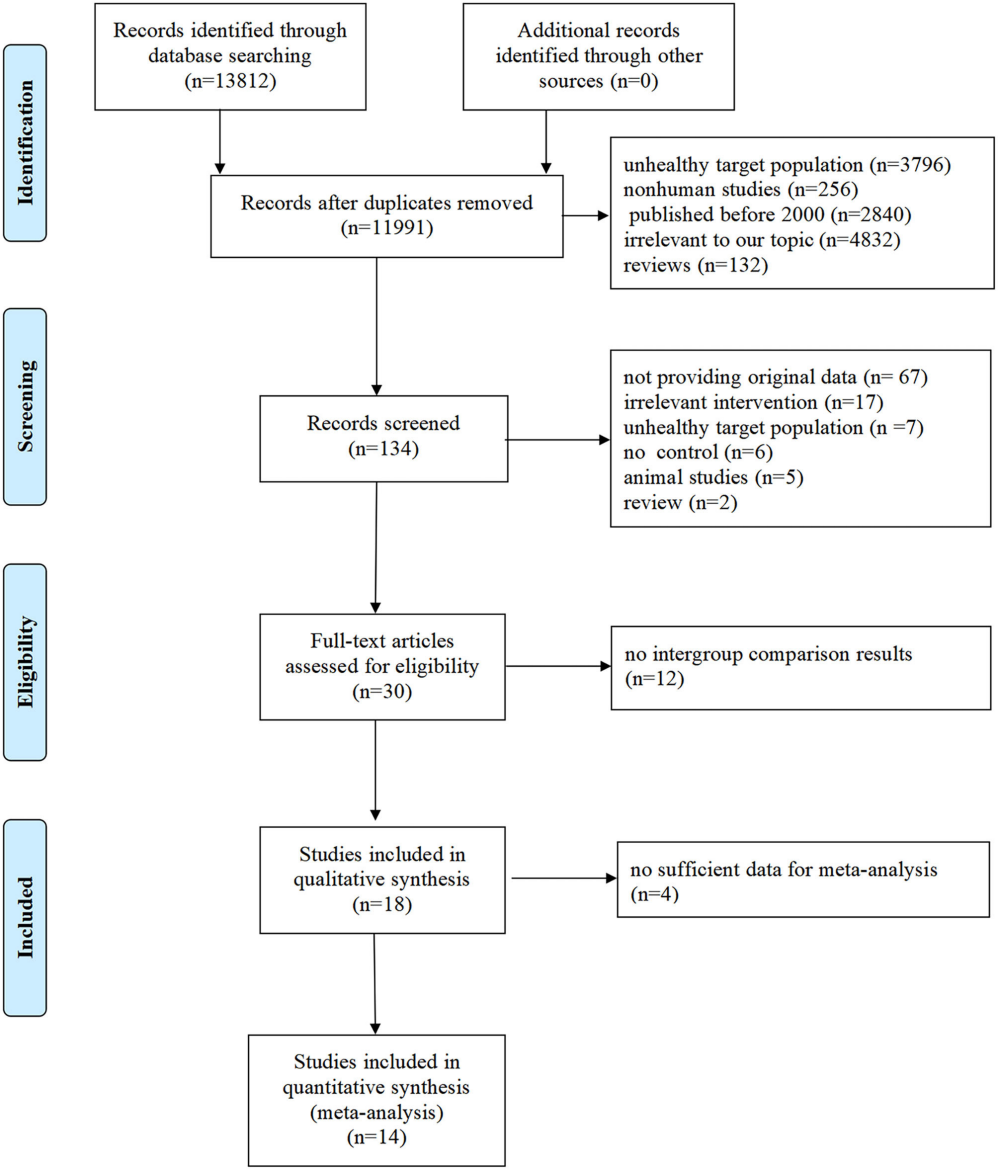
Flowchart of literature search and selection of studies.

A total of 13,812 potentially relevant articles were found in the initial search, and 11,991 articles were excluded by removing the duplications and screening the titles and/or abstracts. Full-text evaluations were conducted for the remaining 134 articles, and 104 of these articles were excluded for not meeting inclusion criteria. Eventually, 18 articles were included in our analysis (29–46). The details of the step-by-step trial's identification and selection process are given in Figure 1.

### Characteristic of Included Studies and Subjects

The effects of carotenoids were evaluated by oxidative stress parameters, carotenoid level, and lipids or lipoprotein levels. Trials were conducted in 9 countries and published between 2000 and 2017. The sample size ranged from 12 to 99 subjects and age ranged from 10.0 to 71.77 years old. BMI ranged from 18 to 27.8 kg/m^2^ and weight ranged from 20.2 to 82.3 kg. The target populations were healthy subjects in 16 studies, athletes in 1 study (29), and pregnant women in 1 study (30). Table 1 presents the fundamental information of all the subjects.

**Table 1:** Fundamental information of subjects.

**References**	**Country**	**Sex (male/female)**		**Age (years)**		**Body mass index (kg/m^**2**^)**		**Weight (kg)**	
		**Treatment (T)**	**Control (C)**	**Treatment (T)**	**Control (C)**	**Treatment (T)**	**Control (C)**	**Treatment (T)**	**Control (C)**
Duthie et al. (29)	UK	9/12	10/14	48.3 (5.6)	48.5 (4.8)	26.6 (3.9)	26.0 (3.4)	–	
Ryu et al. (30)	Korea	22/11	22/8	48.2 (1.4)	50.9 (1.6)	23.9 (0.6)	24.5 (0.5)	–	
Turner et al. (31)	USA	0/34	0/33	23.0 (4.0)	24.0 (5.0)	20.4 (2.9)		20.2 (2.3)	45.4 (6.5)
Miyazawa et al. (32)	Japan	3/3	4/2	58.0 (7.0)	57.0 (7.0)	23.4 (3.4)	22.5 (2.5)	61.4 (11.5)	59.3 (10.4)
Ma et al. (33)	China	—	—	T1: 10.0 (0.6); T2: 18.6 (1.2); T3: 65.3 (9.0)	C1: 10.1 (0.7); C2: 18.6 (0.8); C3: 65.3 (7.3)	T2: 21.2 (2.0); T3: 23.7 (3.2)	C2: 21.1 (2.8); C3: 24.4 (3.2)	T1: 31.4 (4.8); T2: 59.5 (9.0); T3: 61.9 (9.9)	C1: 33.6 (6.5); C2: 60.5 (10.1); C3: 63.6 (8.6)
Jacob et al. (34)	Spain	12	12	23.0 (2.0)		21.5 (2.8)		–	
Briviba et al. (35)	Germany	T1: 21/0; T2: 21/0	21/0	T1: 31.0 (9.0); T2: 31.0 (9.0)	32.0 (9.0)	T1: 24.1 (2.4); T2: 23.8 (2.8)	24.3 (3.2)	—	
Concentrate et al. (36)	USA	31	28	25.3 (3.4)	27.4 (8.1)	24.0 (3.9)	23.4 (3.1)	—	
Tauler et al. (37)	Spain	8/0	7/0	23.3 (2.0)		24.5 (1.3)		70.8 (1.2)	
Aust et al. (38)	Germany	T1: 12; T2: 12	12	–		(18.0–25.0)		–	
Hininger et al. (39)	France	0/33	0/32	29.0 (3.4)	28.0 (4.5)	23.3 (74.6)	22.8 (76.9)	—	
Upritchard et al. (40)	Netherlands	T1: 14/19; T2: 13/20	11/20	(35.0–70.0)		(18.0–32.0)		—	
Nelson et al. (41)	USA	T1: 13; T2: 15; T3: 13	14	71.2 (5.5) (65.0–85.0)	27.8 (7.1) (16.6–39.9)	–	
Kiokias and Gordon (42)	UK	31		31.77 (11.3)		22.47 (3.0)		–	
Heinrich et al. (43)	Germany	T1: 12; T2: 12	12	(22.0–55.0)		—		—	
Schmidt et al. (44)	USA	21	19	22.5 (3.9)	22.9 (5.1)	—		80.0 (11.3)	82.3 (8.7)
Stahl et al. (45)	Germany	9	10	(26.0–67.0)		—		—	
Stahl et al. (46)	Germany	10	10	(20.0–57.0)		—		—	

The treatment durations are mainly 4–12 weeks, with the shortest duration being 2 weeks and the longest duration being 12 weeks. The tested carotenoids in blood were β-carotene, α-carotene, lutein, lycopene, zeaxanthin, and α-tocopherol. The interventions of 14 trials were carotenoids complex, 4 trials were fruits/vegetables, and 0 trial was single carotenoid. The formulations of carotenoids were capsules or tablets and fruits/vegetables were juice or servings. The doses of carotenoids complex were characterized into low dose (<20 mg), medium dose (≥20 mg, <50 mg), and high dose (>50 mg). Among the studies for meta-analysis, both the low and medium doses of carotenoids complex were applied in trials measuring HDL and TG and, thus, subgroup analyses stratified by doses were conducted, simple medium dose of carotenoids complex was seen in trials with FRAP measurement, and simple low doses were found in trials with other measured parameters. Of all included studies, only one study applied high doses of carotenoids complex and did not participate in meta-analysis as the necessary data were unavailable. Table 2 shows the detailed interventions and measurements of all the included studies.

**Table 2:** Intervention and measurement parameters of all included studies classified by intervention type.

**References**	**Treatment (T)**	**Control (C)**	**Measurement**
**Carotenoids complex**			
Ryu (30)	5.0 g chlorella (13.0 mg lutein, 0.25 mg zeaxanthin, 1.2 mg α-carotene, 0.85 mg β-carotene) (daily, 4 weeks)	Placebo (daily, 4 weeks)	TG (μmol/L), HDL (μmol/L)
Miyazawa (32)	8.0 g chlorella (22.9 mg lutein, 5.0 mg β-carotene) (daily, 2 months)	0.0 g chlorella (daily, 2months)	HDL (mg/dL), TG (mg/dL), lutein (μmol/L), zeaxanthin μmol/L), α-carotene (μmol/L), β-carotene (μmol/L), lycopene (μmol/L), α-tocopherol (μmol/L)
Ma (33)	T1 (children): 2,000.0 IU retinol, 1.0 mg β-carotene, 100.0 mg α-tocopherol, 300.0 mg ascorbic acid, 200.0 μg selenium (daily, 2 months); T2 (young people): 3,000.0 IU retinol, 1.5 mg β-carotene, 200.0 mg α-tocopherol, 500.0 mg ascorbic acid, 400.0 μg selenium (daily, 2 months); T3 (old people): 3,000.0 IU retinol, 1.5 mg β-carotene, 200.0 mg α-tocopherol, 500.0 mg ascorbic acid, 400.0 μg selenium (daily, 2 months)	C1 (children): placebo (daily, 2 months); C2 (young people): placebo (daily, 2 months); C3 (old people): placebo (daily, 2 months)	β-carotene (μmol/L), tocopherol (μmol/L)
Concentrate (36)	7.5 mg β-carotene, 234.0 mg vitamin C, 45.0 IU vitamin E, 420.0 mg folate, 60.0 mg calcium (daily, 11 weeks)	Placebo (microcrystalline cellulose) (daily, 11 weeks)	ORAC (μmol/L), β-carotene (μmol/L), lutein (μmol/L), lycopene (μmol/L)
Tauler (37)	250.0 mg Vitamin E, 15.0 mg of β-carotene (daily, 90 days), 1.0 g vitamin C (daily, 16–90 days)	Placebo (lactose) (daily, 90 days)	SOD (pKat/109 cells), CAT (K/109 cells), GPx (nKat/109 cells)
Aust (38)	T1: 9.8 mg lycopene, 0.8 mg phytofluene, 1.0 mg phytoene, 0.4 mg β-carotene (daily, 12 weeks); T2: 8.2 mg lycopene, 3.2 mg phytofluene, 4.6 mg phytoene, 0.4 mg β-carotene daily, 12 weeks)	10.2 mg lycopene, 0.0 mg phytofluene, 0.0 mg phytoene, 0.0 mg β-carotene (daily, 12 weeks)	lycopene (μmol/L), β-carotene (μmol/L)
Hininger (39)	60.0 mg Vitamin C, 4.8 mg β-carotene, 10.0 mg vitamin E, 1.4 mg thiamin, 1.6 mg riboflavin, 15.0 mg niacin, 6.0 mg pantothenic acid, 200.0 mg folic acid, 1.0 mg cobalamin, 15.0 mg Zn, 87.5 mg Mg, 100.0 mg Ca (daily, 2months)	Placebo (daily, 2 months)	β-carotene (μmol/L)
Upritchard (40)	T1: 43.0 mg vitamin E, 0.22 mg lutein, 0.06 mg lycopene, 0.06 mg α -carotene, 0.11 mg β -carotene (daily, 11 weeks) T2: 111.0 mg vitamin E, 0.63 mg lutein, 0.18 mg lycopene, 0.14 mg α -carotene, 0.28 mg β -carotene (daily, 11 weeks)	1.3 mg vitamin E (daily, 11 weeks)	HDL(μmol/L), TG (μmol/L), α-tocopherol (μmol/L), lutein (μmol/L), α-carotene (μmol/L), β-carotene (μmol/L), lycopene (μmol/L), FRAP (μmol/L)
Nelson (41)	T1: one food group (11.0 mg β-carotene, 6.0 mg lutein, 0.6 mg zeaxanthin, 10. 0 mg lycopene) (daily, 5 weeks) T2: 2 antioxidant capsule (2.4 mg β-carotene, 6 mg lutein/zeaxanthin, 0.5 mg lycopene) (daily, 5 weeks) T3: 2 antioxidant tablet (4.0 mg β-carotene, 4.0 mg lutein/zeaxanthin) (daily, 5 weeks)	Placebo (daily, 5 weeks)	lutein (μmol/L), zeaxanthin (μmol/L), lycopene (μmol/L), α-carotene (μmol/L), β-carotene (mmol/L), α-tocopherol (μmol/L), ORAC (μmol/L)
Kiokias (42)	4 capsules (1.0 g) (fish oil), 24.6 mg tomato extract, 6.3 mg palm oil carotene extract, 2.0 mg marigold extract, 3.7 mg paprika extract, 3.7 mg bixin (daily, 6 weeks)	4 capsules (1g) (fish oil) (daily, 6 weeks)	TG (μmol/L), HDL (μmol/L), ORAC (mM)
Heinrich (43)	T1: 24.0 mg β-carotene, soybean oil (daily, 12 weeks) T2: 8.0 mg β-carotene, 8.0 mg lycopene, 8.0 mg lutein, soybean oil (daily, 12 weeks)	Soybean oil (daily, 12 weeks)	β-carotene (μmol/L), lutein (μmol/L), lycopene (μmol/L)
Schmidt (44)	20,050.0 IU β-carotene, 330.0 mg ascorbic acid, 650.0 IU α-,β-,γ-, δ-tocopherols, 167.0 g selenium, 13.2 mg catechin, 500.0 μg lutein, 100.0 μg lycopene, 181.0 mg N-acetyl 1-cysteine, 5.0 mg pomegranate extract, 100.0 mg vegetable blend concentrate (lutein, β-carotene, α-carotene, lycopene) (daily, 24 days)	Placebo (daily, 24 days)	FRAP (moles trolox equivalents/ml), ORAC (moles trolox Equivalents/ml), α-carotene (μg/ml), β-carotene (μg/ml), lutein (μg/ml), lycopene (μg/ml), α-tocopherol (μg/ml), zeaxanthin (μg/ml)
Stahl (45)	40.0 g tomato paste, 10.0 g olive oil, 16.0 mg lycopene, 0.5 mg β-carotene 0.1 mg lutein (daily, 10 weeks)	10.0 g olive oil (daily, 10 weeks)	α-tocopherol (μmol/L), α-carotene (μmol/L), β-carotene (μmol/L), lutein (μmol/L), lycopene (μmol/L)
Stahl (46)	Carotenoid, vitamin E (daily, 12 weeks)	25.0 mg total carotenoids, 25.0 mg carotenoids, 13.0 mg all-trans-β-carotene, 10.5 mg 9-cis β-carotene, 0.3 mg other cis isomers β-carotene, 0.75 mg α-carotene, 0.18 mg cryptoxanthin, 0.15 mg zeaxanthin, 0.12 mg lutein (daily, 12 weeks)	β-carotene (μmol/L), α-tocopherol (μmol/L)
**Fruits/vegetables**			
Duthie (29)	480.0 g diverse fruits, vegetables, fruit juices (daily, 12 weeks)	3 or fewer portions fruits and vegetables (daily, 12 weeks)	FRAP (μmol Fe(II)/L), SOD (U/g Hb), CAT (U/g Hb), GPx (U/g Hb), HDL (μmol/L), TG μmol/L) α-tocopherol (μg/ml), β-carotene (μg/ml), α-carotene (μg/ml), lycopene (μg/ml)
Turner (31)	0.5 mg vitaminA, 200.0 g orange-fleshed sweet potatoes (daily, 6 days/week, 3 weeks)	200.0 g white-fleshed sweet potatoes, 2 corn-oil capsule (daily, 6 days/week, 3 weeks)	β-carotene (μmol/L), α-carotene (μmol/L), lycopene (μmol/L), α-tocopherol (μmol/mmol lipid)
Jacob (34)	500.0 ml tomato juice (41.8 mg lycopene, 90.0 mg vitamin C) (daily, 2 weeks)	500.0 ml tomato juice (870.0 mg vitamin C) (daily, 2 weeks)	lycopene (μmol/L), β-carotene (μmol/L), lutein (μmol/L), tocopherol (mg/l), FRAP (μmol/L)
Briviba et al. (35)	T1: 0.8 (0.2) servings vegetables, 1.1 (0.4) servings fruits [total 1.9 (0.5) servings] (daily, week 1–4) 4.3 (0.6) servings vegetables, 3.5 (0.5) servings fruits [total 7.8 (1.1) servings] (daily, weeks 4–8); T2: 0.9 (0.2) servings vegetables, 1.0 (0.3) servings fruits [total 1.9 (0.4) servings] (daily, week 1–4) 2.8 (0.4) servings vegetables, 1.9 (0.3) fruits servings [total 4.6 (0.7) servings] (daily, weeks 4–8)	1.0 (0.3) servings vegetables, 1.0 (0.4) servings fruits [total 2.0 (0.6) servings] (daily, week 1–4) 1.0 (0.5) servings vegetables, 1.0 (0.4) servings fruits [total 2.0 (0.8) servings] (daily, weeks 4–8)	Lutein (nM), zeaxanthin (nM), α-carotene (nM), β-carotene (nM), lycopene (nM)

*ORAC, oxygen radical absorbance capacity; TG, triglyceride; HDL, high density lipoprotein; FRAP, ferric-reducing ability of plasma; SOD, superoxide dismutase; CAT, catalase*.

### Quality Assessment

**Figure 2:**
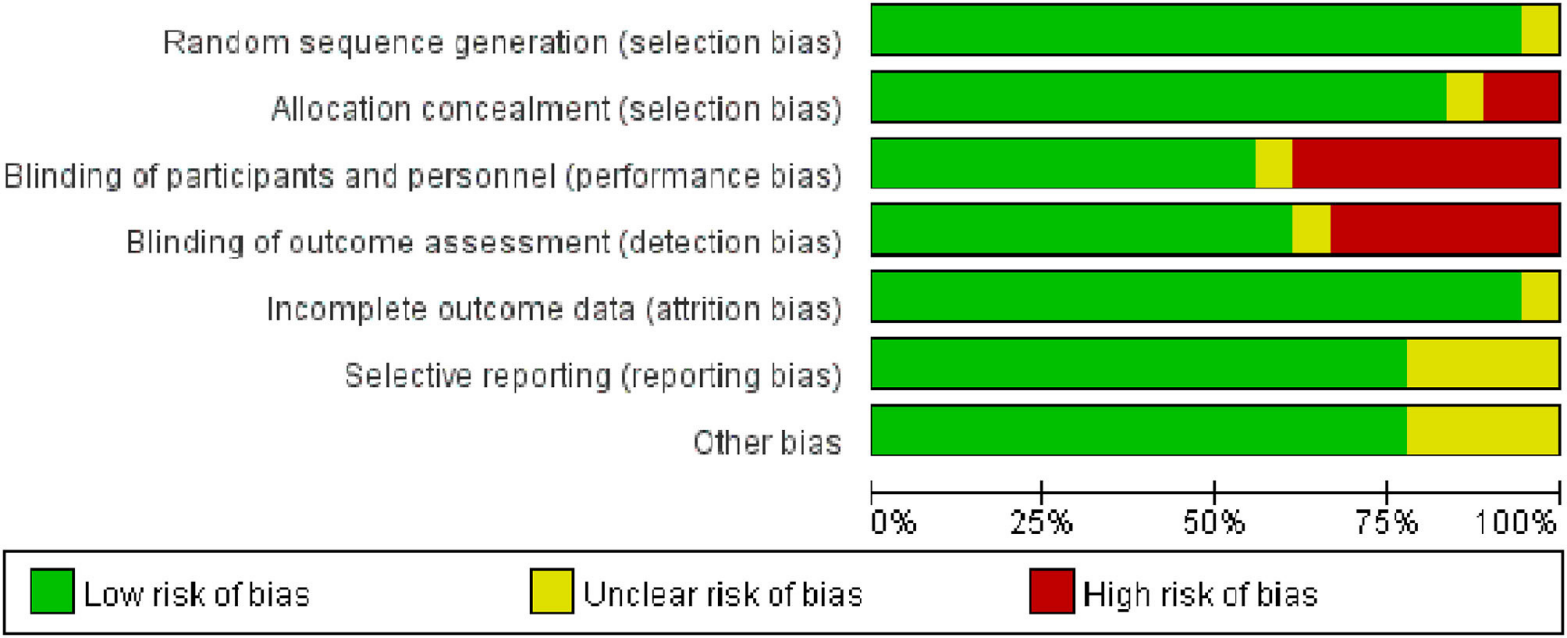
The methodological quality assessment of included studies based on risk of biases presenting as percentages across all included studies.

**Figure 3:**
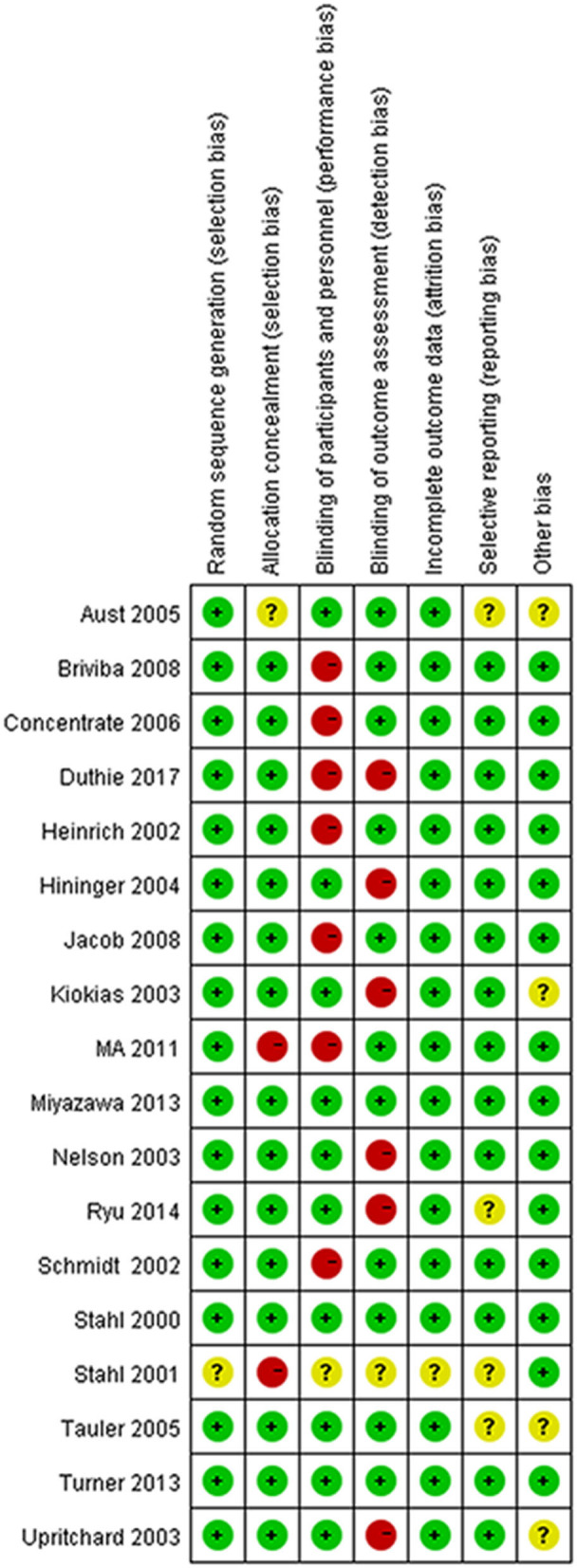
The detailed results of risk of bias in each included study.

All the trials except for 1 trial (32) used an appropriate method of random sequence generation. The majority of trials (15/18) were assessed as low risk of bias for allocation concealment. One trial did not explain the detailed process of blinding, 10 trials were classified as low risk of blinding of participants and personnel (performance bias) and 11 trials had a low risk of blinding of outcome assessment (detection bias). One trial explained the unclear risk of incomplete outcome data (attrition bias). Risks of bias are shown in Figures 2, 3.

### Meta-Analysis Results and Descriptive Analysis Results

#### Two Antioxidative Capability Parameters

**Figure 4:**
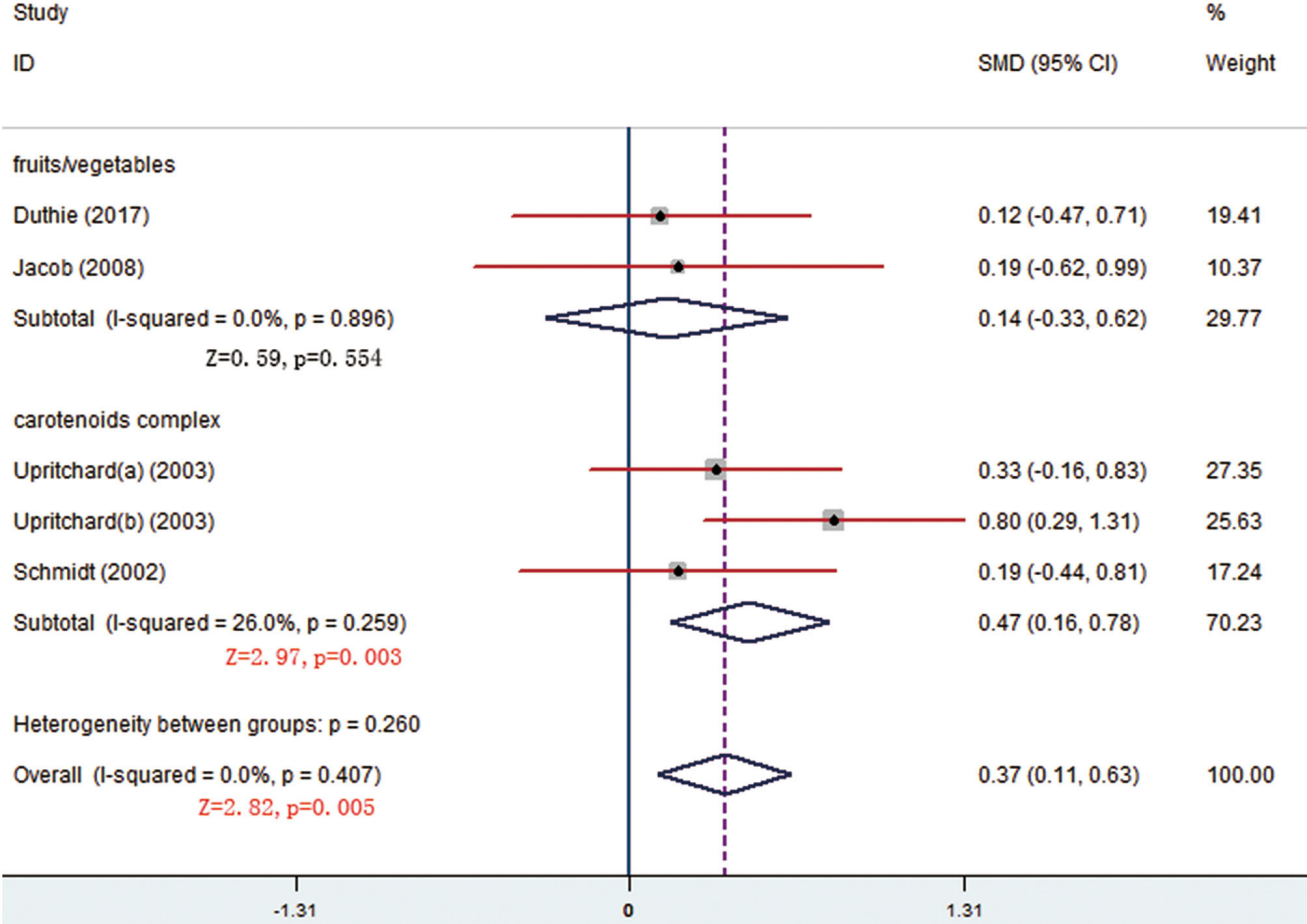
Meta-analysis and subgroup analysis showing that carotenoids complex significantly increased ferric-reducing ability of plasma (FRAP) comparing to control group.

**Figure 5:**
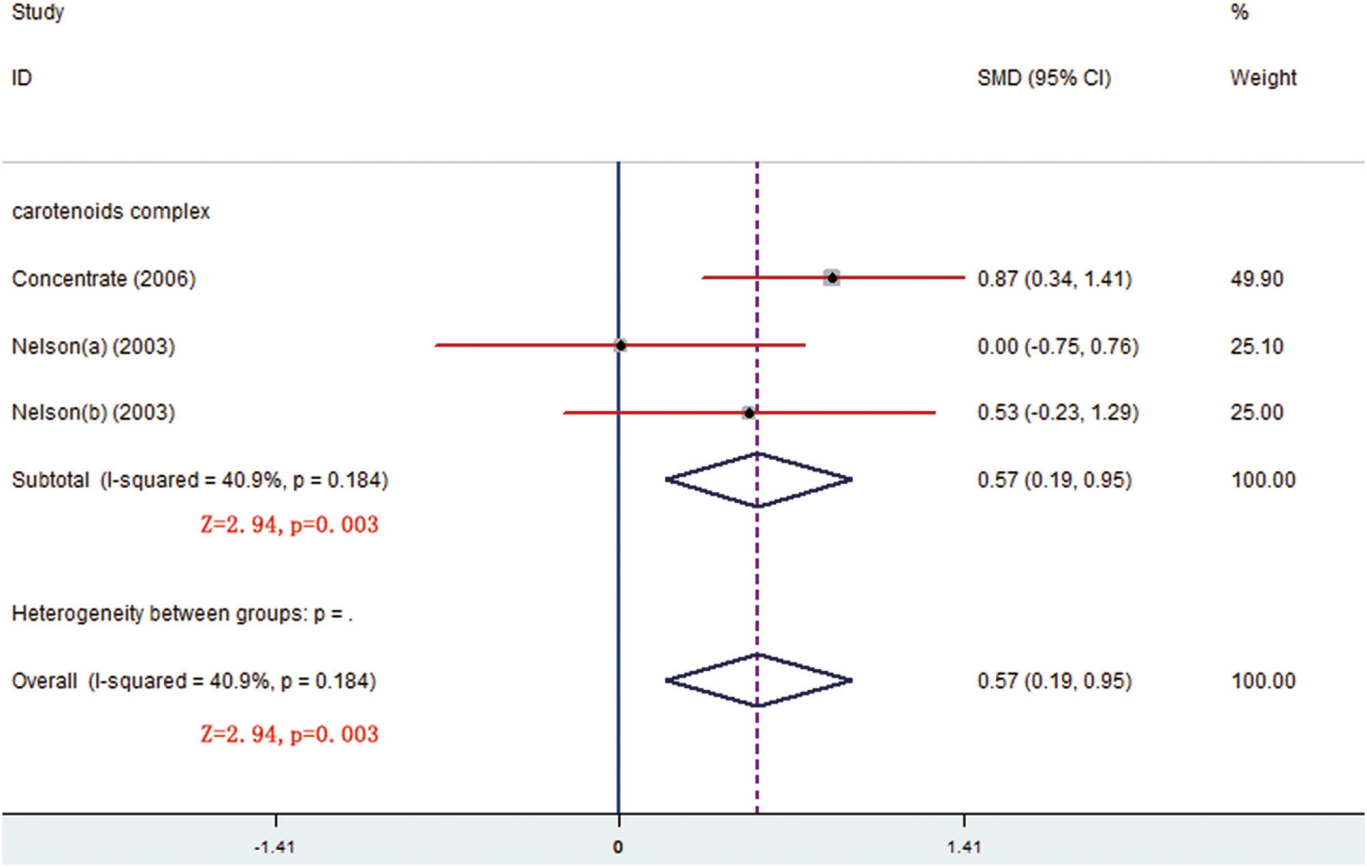
Meta-analysis result showing that carotenoids complex significantly increased oxygen radical absorbance capacity (ORAC) concentration comparing to control group.

There were respective 5 and 3 trials that compared change levels of FRAP and ORAC between the treatment and control groups. Both the FRAP and ORAC concentrations were significantly increased in the group receiving carotenoids compared with the control groups (SMD = 0.371; 95% CI: 0.113–0.629, *p* = 0.005; SMD = 0.568; 95% CI: 0.190–0.947, *p* = 0.003) (Figures 4, 5). Subgroup analysis of FRAP showed that medium-dose carotenoids complex significantly increased antioxidative capability comparing to control (SMD = 0.468; 95% CI: 0.159–0.776, *p* = 0.003), while supplements of fruits/vegetables did not significantly increased antioxidative capability (*p* > 0.05) (Table 3).

**Table 3:** Meta-analysis/subgroup analysis and publication bias of included studies.

**Measurement index**		**Number of study**	**SMD**	**95% CI**		***p***	**Heterogeneity**		**Model**	**Publication bias**	
							***I* ^2^**	***p***		***t***	***p***
**Antioxidative capability parameters**										
FRAP	Overall	5	0.371	0.113	0.629	0.005^*^	0.00%	0.407	Fixed	−1.06	0.368
	Fruits/vegetables	2	0.143	−0.331	0.616	0.554	0.00%	0.896	Fixed		
	Carotenoids complex (MD)	3	0.468	0.159	0.776	0.003^*^	26.0%	0.259	Fixed		
ORAC	carotenoids complex (LD)	3	0.568	0.190	0.947	0.003^*^	40.9%	0.184	Fixed	−1.61	0.354
**Antioxidative enzymes**										
SOD	Overall	3	0.237	−0.673	1.147	0.200	67.1%	0.048	Random	0.55	0.680
	Fruits/vegetables	1	−0.043	−0.629	0.542	0.885	–	–	–		
	Carotenoids complex (LD)	2	0.458	−1.320	2.237	0.614	81.3%	0.021	Random		
CAT	Overall	3	0.173	−0.285	0.631	0.459	0.5%	0.366	Fixed	0.72	0.604
	Fruits/vegetables	1	0.036	−0.550	0.621	0.905	–	–	–		
	Carotenoids complex (LD)	2	0.390	−0.346	1.125	0.299	31.8%	0.226	Fixed		
GPx	Overall	3	1.369	−0.324	3.062	0.117	87.0%	0.000	Random	4.84	0.130
	Fruits/vegetables	1	0.046	−0.539	0.632	0.877	-	-	-		
	Carotenoids complex (LD)	2	2.180	−0.112	4.472	0.062	81.1%	0.021	Random		
**Carotenoid levels**										
β-carotene	Overall	11	0.294	−0.098	0.687	0.142	67.6%	0.001	Random	0.85	0.416
	Fruits/vegetables	6	0.189	−0.227	0.605	0.373	64.7%	0.015	Random		
	Carotenoids complex (LD)	5	0.496	−0.399	1.392	0.277	74.7%	0.003	Random		
	Week 4	5	−0.168	−0.526	0.190	0.357	20.6%	0.283	Random		
	Week 8	4	0.490	0.123	0.858	0.009^*^	0.00%	0.451	Random		
	Fruits/vegetables	2	0.665	0.232	1.098	0.003^*^	0.0%	0.593	Random		
	Carotenoids complex (LD)	2	0.042	−0.652	0.736	0.906	0.0%	0.727	Random		
	Week 12	2	1.281	−0.435	2.996	0.143	88.0%	0.004	Random		
α-carotene	Overall (week 4)	4	0.322	−0.011	0.655	0.058	0.0%	0.837	Fixed	2.10	0.171
	Fruits/vegetables	1	0.400	−0.192	0.992	0.185	–	–	–		
	Carotenoids complex (LD)	3	0.286	−0.118	0.689	0.165	0.0%	0.686	Fixed		
lutein	Overall	5	0.756	−0.062	1.575	0.070	80.0%	0.000	Random	2.38	0.097
	Carotenoids complex (LD)	3	1.325	−0.297	2.947	0.109	85.8%	0.001	Random		
	Fruits/vegetables	2	0.138	−0.291	0.566	0.528	0.0%	0.627	Random		
	Week 4	3	0.391	−0.262	1.043	0.241	53.8%	0.115	Random		
	Week 12	2	1.229	−1.235	3.693	0.328	92.5%	0.000	Random		
Lycopene	Overall	15	0.303	−0.129	0.736	0.169	83.4%	0.000	Random	−0.28	0.783
	Fruits/vegetables	8	0.202	−0.276	0.681	0.408	84.2%	0.000	Random		
	Carotenoids complex (LD)	7	0.611	−0.372	1.595	0.223	84.9%	0.000	Random		
	Week 4	7	0.430	−0.306	1.166	0.252	82.8%	0.000	Random		
	Week 8	8	0.258	−0.279	0.795	0.346	83.7%	0.000	Random		
Zeaxanthin	Overall (week 4) carotenoids complex (LD)	3	0.249	−0.153	0.651	0.225	0.0%	0.936	Random	2.04	0.290
α-tocopherol	Overall	6	0.752	0.020	1.485	0.044^*^	75.2%	0.001	Random	4.01	0.016^*^
	Fruits/vegetables	2	−0.102	−0.530	0.326	0.641	0.0%	0.760	Random		
	Carotenoids complex (LD)	4	1.314	0.520	2.107	0.001^*^	48.5%	0.120	Random		
	Week 4	3	1.304	−0.327	2.936	0.117	86.80%	0.001	Random		
	Week 8	3	0.284	−0.351	0.919	0.381	39.20%	0.193	Random		
**Lipids or lipoproteins**										
HDL	Overall	5	0.061	−0.191	0.312	0.635	41.4%	0.146	Fixed	−0.51	0.642
	Carotenoids complex	4	0.132	−0.146	0.410	0.352	44.9%	0.142	Fixed		
	Carotenoids complex (LD)	2	0.102	−0.248	0.452	0.569	79.2%	0.028	Fixed		
	Carotenoids complex (MD)	2	0.184	−0.274	0.641	0.431	0.0%	0.455	Fixed		
	Fruits/vegetables	1	−0.258	−0.846	0.330	0.390	–	–	–		
TG	Carotenoids complex	3	−0.410	−0.698	−0.122	0.005^*^	0.0%	0.405	Fixed	−2.82	0.217
	Carotenoids complex (LD)	2	−0.297	−0.646	0.052	0.095	0.0%	0.460	Fixed		
	Carotenoids complex (MD)	1	−0.652	−1.163	−0.140	0.013^*^	–	–	–		

*FRAP, ferric-reducing ability of plasma; ORAC, oxygen radical absorbance capacity; SOD, superoxide dismutase; CAT, catalase; GPx, glutathione peroxidase; HDL, high density lipoprotein; TG, total glyceride; LD, low dose (<20 mg); MD, medium dose (≥20 mg, <50 mg)*.

* *Significantly different (p < 0.05)*.

#### Three Antioxidative Enzymes Parameters

The changes from posttreatment to the baseline of SOD, CAT, and GPx were compared between treatment and control groups. No significant differences were seen for all the parameters (*p* > 0.05) neither pooled results nor subgroup results were stratified by different forms of oral carotenoids (Table 3).

#### Blood Levels of Seven Carotenoid Levels Indexes

**Figure 6:**
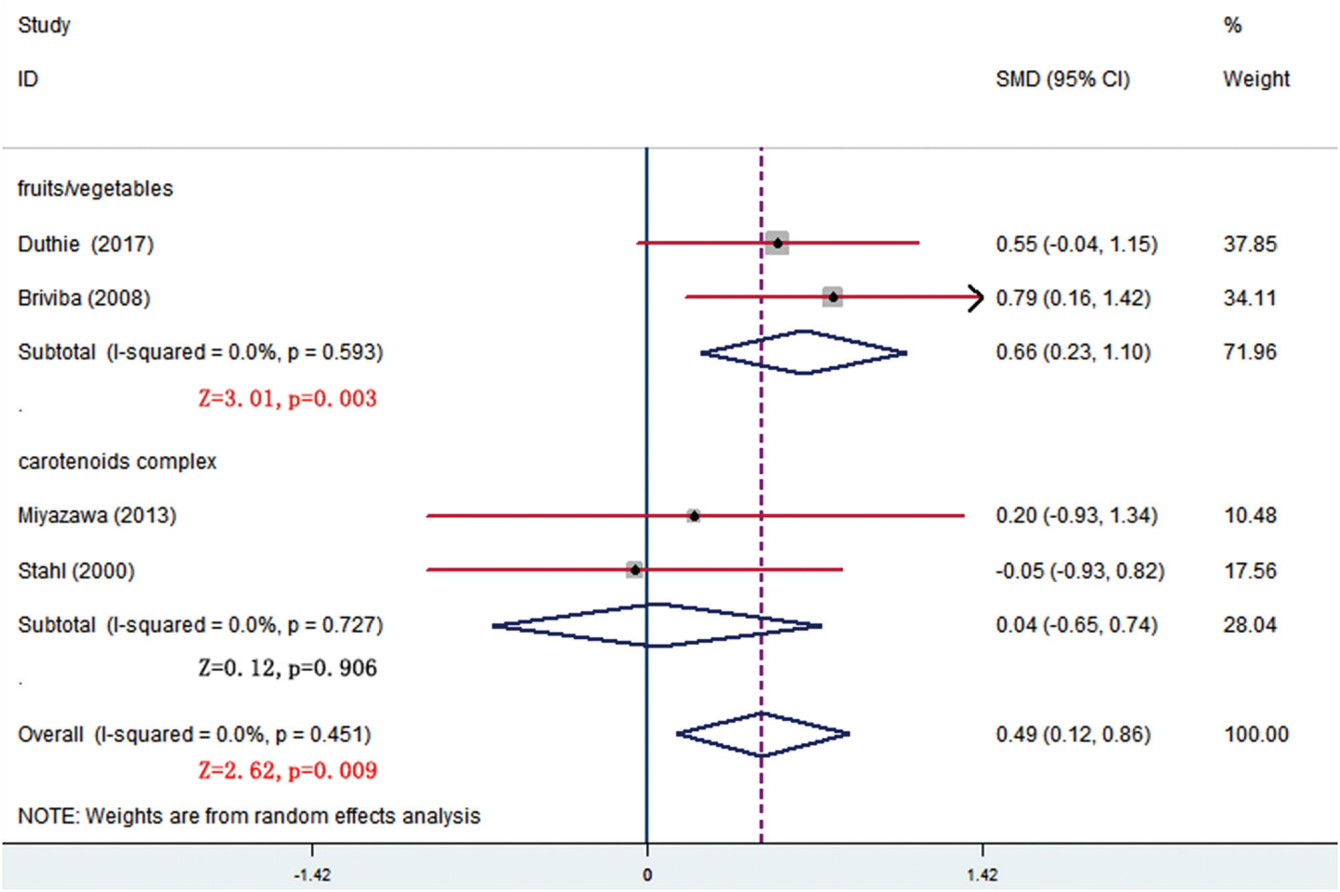
Subgroup analysis of β-carotene showing a higher β-carotene level in fruits/vegetables group than in placebo group at week 8.

In all, 14 trials compared β-carotene levels between the treatment and control groups. Meta-analysis of 11 trials showed that significant difference was seen at week 8 and the difference came from fruits/vegetables (SMD = 0.665, 95% CI: 0.232–1.098, *p* = 0.003) (Figure 6), as the fruits/vegetables treatment group produced higher β-carotene level than the control group (Table 3). The other 3 trials that had no deserved data for meta-analysis also showed that the treatment groups had higher β-carotene levels than the control groups (*p* = 0.003; Table 4).

**Table 4:** Descriptive analyses of included studies that had no sufficient data for meta-analyses.

**Measurement index**	**Intervention**	**References**	**Outcome**
**β-carotene**			
	Fruits/vegetables	Turner (31)	Plasma β-carotene increased 250% in the orange-fleshed sweet potatoes group, and the mean change in plasma β-carotene (0.306 ± 0.070 mmol/L) was different from that in the white-fleshed sweet potatoes group (*p* < 0.001).
	Carotenoids complex (HD)	Ma (33)	Significant increases of plasma β-carotene after 2 months supplementation in all three supplemented groups compared with the respective placebo groups (*p* < 0.001).
	carotenoids complex (LD)	Hininger (39)	Significant higher level of plasma β-carotene in the supplemented group at 9 months of gestation, comparing to placebo group (*p* < 0.05).
**α-carotene**			
	Carotenoids complex (LD)	Ryu (30)	Remarkable changes of α-carotene between two groups (Chlorella 163.6%; placebo 15%; *p* < 0.0001).
**Lutein**			
	Carotenoids complex (LD)	Ryu (30)	Remarkable changes of lutein two groups (Chlorella 89.6%; placebo −1.7%; *p* < 0.0001).
**Zeaxanthin**			
	Carotenoids complex (LD)	Ryu (30)	Remarkable changes of zeaxanthin between two groups (Chlorella 89.6%; placebo −1.7%; *p* < 0.0001).
**α-tocopherol**			
	Fruits/vegetables	Turner (31)	No significant differences between orange-fleshed sweet potatoes group and white-fleshed sweet potatoes group.
**HDL**			
	Carotenoids complex (LD)	Ryu (30)	Remarkable changes of HDL (Chlorella 4.0%; placebo s betwe*p* = 0.023) compared with placebo.
**TG**			
	Carotenoids complex (LD)	Ryu (30)	Remarkable changes of TG (Chlorella 4.0%; placebo sweet p*p* = 0.023) compared with placebo.

*HDL, high density lipoprotein; TG, triglyceride; LD, low dose (<20 mg); MD, medium dose (≥20 mg, <50 mg); HD, high dose (≥50 mg)*.

**Figure 7:**
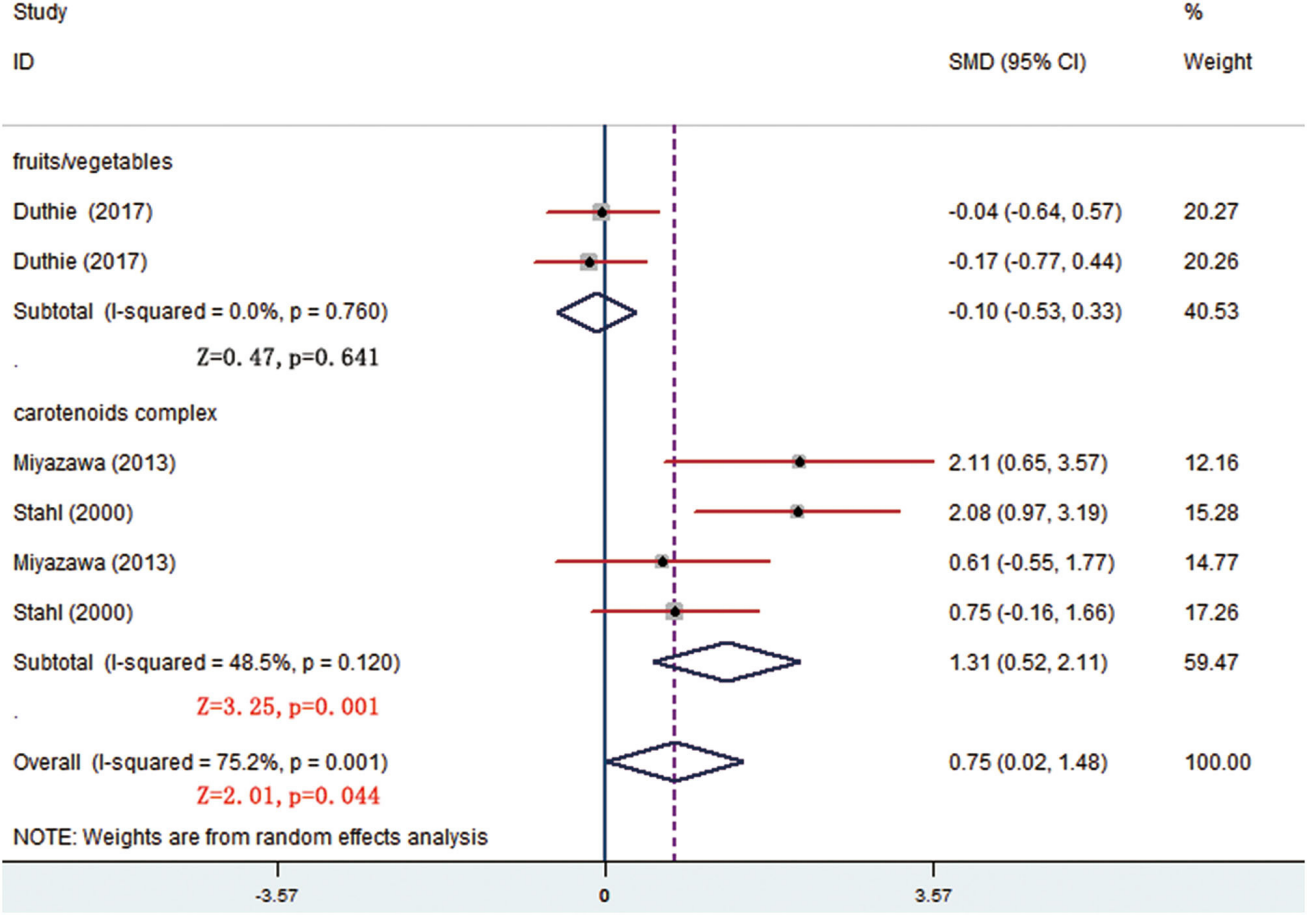
Subgroup analysis of α-tocopherol showing a higher α-tocopherol level in carotenoids complex than in placebo group.

There were 7 trials comparing α-tocopherol levels between the treatment and control groups. The pooled results of 6 trials showed significant differences (*p* < 0.05) and the subgroup analysis presented that supplement of low-dose carotenoids complex contributed to the difference (SMD = 1.314, 95% CI: 0.520–2.107, *p* = 0.001) (Figure 7) (Table 3). One trial showed that no significant differences were present between the orange-fleshed sweet potatoes treatment group and the white-fleshed sweet potatoes control group (*p* > 0.05; Table 4).

The meta-analyses results showed that the levels of other 5 kinds of carotenoid, i.e., α-carotene, lycopene, lutein, zeaxanthin, and β-cryptoxanthin were not different between treatment and control groups (*p* > 0.05) (Table 3). One trial that had no sufficient data for meta-analyses showed that remarkable changes of α-carotene, lutein, or zeaxanthin were, respectively, seen between the low-dose carotenoids complex group and the placebo group (*p* < 0.05; Table 4).

#### Lipid or Lipoprotein Parameters

**Figure 8:**
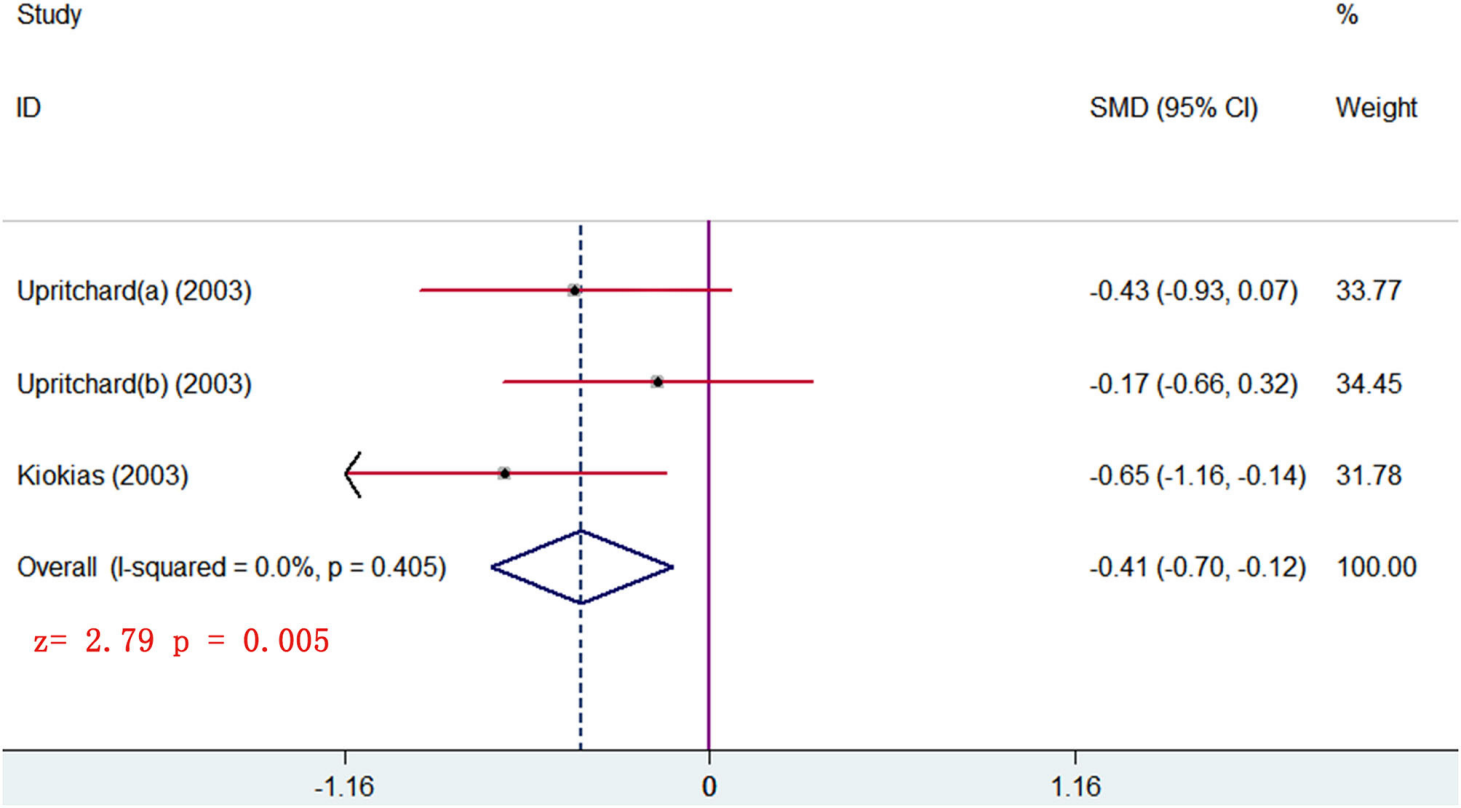
subgroup analysis of TG level showing supplements of carotenoids complex significantly decreased the TG level in blood.

The changes from posttreatment to the baseline of HDL and TG were compared between treatment and control groups. The pooled results showed that no significant differences were seen of any parameters (*p* > 0.05). The subgroup analysis, stratified by different forms of carotenoids, showed that medium-dose carotenoids complex significantly decreased the blood TG level (SMD = −0.410, 95% CI: −0.698 to −0.122, *p* = 0.005) (Figure 8) (Table 3). The trial that had no sufficient data for meta-analyses showed that remarkable changes of HDL or TG were, respectively, seen between the low-dose carotenoids complex group and placebo group (*p* < 0.05; Table 4).

### Publication Bias (Table 3)

Egger's regression tests showed that most indexes had no evidence of publication bias (*p* > 0.05), with the exception of α-tocopherol (*t* = 4.01, *p* = 0.016) and TG (*t* = −5.89, *p* = 0.004) (Table 3).

## Discussion

A systematic review conducted in 2015 assessed that a range of single carotenoids among which β-carotene and lycopene were the most abundant consumed carotenoids, and β-carotene and α-carotene were the most commonly assessed carotenoids biochemically (47). In recent years, carotenoids complex and dietary botanical carotenoids (especially in fruits/vegetables) were more welcomed than a single form of carotenoid. The current systematic review, not only an updated systematic review of the previous one but specially reported carotenoids complex and dietary botanical carotenoids rather than single carotenoids. Besides, we added antioxidative markers and lipid/lipoprotein parameters that may be influenced by oral carotenoids, and these important markers were not analyzed in the previous systematic review.

In the aspect of antioxidative effects, FRAP and ORAC are standardized indexes measuring the antioxidant capacity of nutraceutical or dietary supplement industries (48). FRAP follows a single electron transfer mechanism and ORAC follows a hydrogen atom transfer mechanism. Antioxidant phenols directly interact with free radicals by electron or hydrogen atom donation to fulfill antioxidative capacity (49). For example, Shanely et al. (50) showed that watermelon puree was associated with increases in plasma antioxidant capacity measured by FRAP and ORAC. In our meta-analysis, pooled results showed that oral carotenoids supplementation significantly increased the blood levels of FRAP, and subgroup analysis indicated that the antioxidative capacity attributed to carotenoids complex but not fruits/vegetables. The pooled results of studies measuring ORAC also showed higher antioxidative capacity after intaking the carotenoids complex. One previous uncontrolled study demonstrated that levels of single antioxidants in food do not necessarily reflect their total antioxidant capacity which also depends on the synergic and redox interactions among the different molecules present in the food (51). This theory may explain the relatively high antioxidative capacity of the carotenoids complex. In the current study, we also assessed the levels of antioxidative enzymes in blood before and after carotenoid supplements. The antioxidative enzymes play protective effects against active and massive oxidative attacks due to the ability to decompose ROS (48). For example, SOD can catalyze superoxide into oxygen and hydrogen peroxide (52), and CAT and GPx can neutralize the hydrogen peroxide by decomposing it into molecular oxygen and water (53, 54). Our meta-analysis showed that oral intake of carotenoids, no matter carotenoids complex or fruits/vegetables, did not influence the blood levels of these antioxidative enzymes. The current results indicated that oral carotenoids play antioxidative roles *via* interacting with free radicals by electron or a hydrogen atom (direct action) but not *via* promoting antioxidative enzymes (indirect action).

Given the relationships between some botanical antioxidants (for example, polyphenol compounds and phytochemicals) and vitagenes network in health benefit (for example, neuroprotection) (3, 55), we should not ignore such potential indirect mechanism of carotenoids. The generation of ROS is involved in the regulation of cellular stress response mechanisms and is a highly regulated process under the control of vitagenes (55, 56). Vitagenes are redox-sensitive genes coding for redox proteins (55, 56). These proteins control a complex network of intracellular signaling pathways relevant to life span and preservation of cellular homeostasis under stress conditions (3). Hormetic dose responses are mediated by endogenous cellular defense pathways (3). Modulation of endogenous cellular defense mechanisms represents a therapeutic intervention in oxidative stress-related diseases (3). Antioxidants including carotenoids may play their protective role through a hormetic-dependent activation of vitagenes (3).

High-density lipoprotein (HDL) is plasma lipoproteins that are macromolecular assemblies of proteins and lipids (57). A review put forward that high levels of lipid oxidation products in HDL appear to associate with the prevention of atherosclerosis (58). In our meta-analysis, the supplement of various carotenoids did not change the level of HDL in the blood. Interestingly, the blood TG level was significantly decreased by the carotenoids complex. According to a previous report, a high level of TG could aggravate oxidative stress, then drove mucosal inflammation and increased mucosal barrier permeability, thus promoting colitis (59). High TG levels also cause prediabetic neuropathy through oxidative-nitrosative stress (60). These results demonstrated that oral intake of carotenoids, especially carotenoids complex, may have protective effects of some oxidative stress relating diseases *via* decreasing TG levels.

After being orally taken, carotenoids are liberated from the food matrix, absorbed into the bloodstream, and then they will exert biological effects (61). Some factors may influence the bioactivities of carotenoids. For example, different isomers of lycopene have discriminatory bioaccessibility. The cis-isomer in tissues exceeds that in foods, and the greater bioaccessibility contributes to the enrichment in tissues, as compared with all-trans isomers (62). Besides, serum concentrations of most carotenoids decrease abiding by first-order kinetics, with plasma half-life between 26 and 76 days (about 4 and 10 weeks) (63). In our systematic review, the blood levels of 6 carotenoids were tested, and α-carotene, β-carotene, and α-tocopherol got positive findings. The blood β-carotene level was not increased until 8 weeks of fruits/vegetable supplement. In addition, the carotenoids complex supplement produced a high level of α-tocopherol after week 4. These results reflect that the intake duration of carotenoids should be long enough to reach enough concentration for function.

This systematic review has several advantages. Different from other systematic reviews, the current one especially, examined the effects of carotenoid supplementations on oxidative stress *in vivo*. We first demonstrated that the carotenoids complex had more advantages over fruits/vegetables. All potential studies, namely, RCTs, case-controlled trials, and controlled trials, were included to obtain as much information as possible, and the methodological quality of each study was relatively high. Nevertheless, the following limitations need further research. The measurement assessment of some types of carotenoids, such as α-carotene and zeaxanthin, was limited to only one time point. We were unable to state whether carotenoids have different antioxidative effects among subjects with variable physiologic states. Besides, origins (natural or synthetic) and administration methods (for example, chlorella crushed or not?) may influence the effects of carotenoids. We cannot provide the important information owing to unavailability.

In conclusion, this systematic review showed that the carotenoids complex is beneficial for alleviating potential oxidative stress *via* interacting with free radicals or decreasing blood TG levels. The intaking duration of carotenoids should be 8 weeks to reach enough concentration for function. Intake of carotenoids nutrition may have huge potentials for disorders/diseases relating to oxidative stress.

## Data Availability Statement

The raw data supporting the conclusions of this article will be made available by the authors, without undue reservation.

## Author Contributions

CZ: conceptualization and methodology. CZ, JZ, and YD: literature search. CZ, YS, and JY: formal analysis and investigation. CZ and YW: writing—original draft preparation and writing—review and editing. YW: resources. YW, YS, X-HG, and H-DC: supervision. All authors have read and agreed to the published version of the manuscript.

## Funding

This study was supported by the National Natural Science Fund (YW, Grant Number 81972940) and the Liaoning Province National Natural Science Fund (YW, Grant Number 2019-ZD-0763).

## Conflict of Interest

The authors declare that the research was conducted in the absence of any commercial or financial relationships that could be construed as a potential conflict of interest.

## Publisher's Note

All claims expressed in this article are solely those of the authors and do not necessarily represent those of their affiliated organizations, or those of the publisher, the editors and the reviewers. Any product that may be evaluated in this article, or claim that may be made by its manufacturer, is not guaranteed or endorsed by the publisher.

## References

[1] Siti HN Kamisah Y Kamsiah J. The role of oxidative stress, antioxidants and vascular inflammation in cardiovascular disease. Vascul Pharmacol. (2015) 71:40–56. 10.1016/j.vph.2015.03.005 25869516

[2] Odegaard AO Jacobs DR Jr Sanchez OA Goff DC Jr Reiner AP Gross MD. Oxidative stress, inflammation, endothelial dysfunctionand incidence of type 2 diabetes. Cardiovasc Diabetol. (2016) 15:51. 10.1186/s12933-016-0369-6 27013319 PMC4806507

[3] Calabrese V Cornelius C Dinkova-Kostova AT Calabrese EJ Mattson MP. Cellular stress responses, the hormesis paradigm, and vitagenes: novel targets for therapeutic intervention in neurodegenerative disorders. Antioxid Redox Signal. (2010) 13:1763–811. 10.1089/ars.2009.3074 20446769 PMC2966482

[4] Choudhari SK Chaudhary M Gadbail AR Sharma A Tekade S. Oxidative and antioxidative mechanisms in oral cancer and precancer: a review. Oral Oncol. (2014) 50:10–8. 10.1016/j.oraloncology.2013.09.011 24126222

[5] Nourazarian AR Kangari P Salmaninejad A. Roles of oxidative stress in the development and progression of breast cancer. Asian Pac J Cancer Prev. (2014) 15:4745–51. 10.7314/APJCP.2014.15.12.4745 24998536

[6] Saha SK Lee SB Won J Choi HY Kim K Yang GM . Correlation between oxidative stress, nutrition, and cancer initiation. Int J Mol Sci. (2017) 18:1544. 10.3390/ijms18071544 28714931 PMC5536032

[7] Viña J Borras C Abdelaziz KM Garcia-Valles R Gomez-Cabrera MC. The free radical theory of aging revisited: the cell signaling disruption theory of aging. Antioxid Redox Signal. (2013) 19:779–87. 10.1089/ars.2012.5111 23841595 PMC3749699

[8] Balcerczyk A Gajewska A Macierzyn'ska-Piotrowsk E Pawelczyk T Bartosz G Szemraj J. Enhanced antioxidant capacity and anti-ageing biomarkers after diet micronutrient supplementation. Molecules. (2014) 19:14794–808. 10.3390/molecules190914794 25232703 PMC6270881

[9] Kirkwood TB Kowald A. The free-radical theory of ageing–older, wiser and still alive: modelling positional effects of the primary targets of ROS reveals new support. BioEssays. (2012) 34:692–700. 10.1002/bies.201200014 22641614

[10] Speakman JR Selman C. The free-radical damage theory. Accumulating evidence against a simple link of oxidative stress to ageing and life span. BioEssays. (2011) 33:255–9. 10.1002/bies.201000132 21290398

[11] Costa JP Vitorino R Silva GM Vogel C Duarte AC Rocha-Santos T. A synopsis on aging theories, mechanisms and future prospects. Ageing Res Rev. (2016) 29:90–112. 10.1016/j.arr.2016.06.005 27353257 PMC5991498

[12] Tan BL Norhaizan ME. Carotenoids how effffective are they to prevent age-related diseases? Molecules. (2019) 24:1801. 10.3390/molecules24091801 31075966 PMC6539799

[13] Sies H. Oxidative stress a concept in redox biology and medicine. Redox Biol. (2015) 4:180–3. 10.1016/j.redox.2015.01.002 25588755 PMC4309861

[14] Swindells K Rhodes LE. Influence of oral antioxidants on ultraviolet radiation-induced skin damage in humans. Photodermatol Photoimmunol Photomed. (2004) 20:297–304. 10.1111/j.1600-0781.2004.00121.x 15533237

[15] Kattoor AJ Pothineni NVK Palagiri D Mehta JL. Oxidative stress in atherosclerosis. Curr Atheroscler Rep. (2017) 19:42. 10.1007/s11883-017-0678-6 28921056

[16] Gorrini C Harris IS Mak TW. Modulation of oxidative stress as an anticancer strategy. Nat Rev Drug Discov. (2013) 12:931–47. 10.1038/nrd4002 24287781

[17] Milani A Basirnejad M Shahbazi S Bolhassani A. Carotenoids: biochemistry, pharmacology and treatment. Br J Pharmacol. (2017) 174:1290–324. 10.1111/bph.13625 27638711 PMC5429337

[18] Fuller CJ Faulkner H Bendich A Parker RS Roe DA. Effect of beta-carotene supplementation on photosuppression of delayed-type hypersensitivity in normal young men. Am J Clin Nutr. 56:684–90. 10.1093/ajcn/56.4.684 1414968

[19] Nakagawa K KikoT Hatade K Sookwong P Arai H Miyazawa T. Antioxidant effect of lutein towards phospholipid hydroperoxidationin human erythrocytes. Br J Nutr. (2009) 102:1280–4. 10.1017/S0007114509990316 19622187

[20] Lambert LA Koch WH Wamer WG Kornhauser A. Antitumoractivity in skin of skh and sencar mice by two dietary beta-carotene formulations. Nutr Cancer. (1990) 13:213–21. 10.1080/01635589009514063 2111908

[21] Saini RK Nile SH Park SW. Carotenoids from fruits and vegetables: Chemistry, analysis, occurrence, bioavailability and biological activities. Food Res Int. (2015) 76:735–50. 10.1016/j.foodres.2015.07.047 28455059

[22] Alaluf S Heinrich U Stahl W Tronnier H Wiseman S. Dietary carotenoids contribute to normal skin colour and UV photosensitivity. J Nutr. (2002) 132:399–403. 10.1093/jn/132.3.399 11880562

[23] Woodside JV Young IS McKinley MC. Fruit and vegetable intake and risk of cardiovascular disease. Proc Nutr Soc. (2013) 72:399–406. 10.1017/S0029665113003029 24050503

[24] Brouwer IA van Dusseldorp M West CE Meyboom S Thomas CM Duran M . Dietary folate from vegetables and citrus fruit decreases plasma homocysteine concentrations in humans in a dietary-controlled trial. J Nutr. (1999) 129:1135–9. 10.1093/jn/129.6.1135 10356077

[25] Collins AR Harrington VJ Drew J Melvin R. Nutritional modulation of DNA repair in a human intervention study. Carcinogenesis. (2003) 24:511–5. 10.1093/carcin/24.3.511 12663512

[26] Boyle SP Dobson VL Duthie SJ Kyle JA Collins AR. Absorption and DNA protective effects of flavonoid glycosides from an onionmeal. Eur J Nutr. (2000) 39:213–23. 10.1007/s003940070014 11131368

[27] Stea TH Mansoor MA Wandel M Uglem S Frølich W. Changes is predictors and status of homocysteine in young male adults after a dietary intervention with vegetables, fruit and bread. Eur J Nutr. (2008) 47:201–9. 10.1007/s00394-008-0714-y 18521531

[28] Root MM McGinn MC Nieman DC Henson DA Heinz SA Shanely RA . Combined fruit and vegetable intake is correlated with improved inflammatory and oxidant status from a cross-sectional study in a community setting. Nutrients. (2012) 4:29–41. 10.3390/nu4010029 22347616 PMC3277099

[29] Duthie SJ Duthie GG Russell WR Kyle JAM Macdiarmid JI Rungapamestry V . Effect of increasing fruit and vegetable intake by dietary intervention on nutritional biomarkers and attitudes to dietary change: a randomised trial. Eur J Nutr. (2017) 55:1855–72. 10.1007/s00394-017-1469-0 28560503 PMC6060837

[30] Ryu NH Lim Y Park JE Kim J Kim JY Kwon SW . Impact of daily Chlorella consumption on serum lipid and carotenoid profiles in mildly hypercholesterolemic adults: a double-blinded, randomized, placebo-controlled study. Nutr J. (2014) 13:57. 10.1186/1475-2891-13-57 24920270 PMC4066283

[31] Turner T Burri B Jamil KM Jamil M. The effects of daily consumption of beta-cryptoxanthin-rich tangerines and beta-carotene-rich sweet potatoes on vitamin A and carotenoid concentrations in plasma and breast milk of bangladeshi women with low vitamin A status in a randomized controlled trial. Am J Clin Nutr. (2013) 98:1200–8. 10.3945/ajcn.113.058180 24004891

[32] Miyazawa T Nakagawa K Takekoshi H Higuchi O Kato S Kondo M . Ingestion of chlorella reduced the oxidation of erythrocyte membrane lipids in senior japanese subjects. J Oleo Sci. (2013) 11:873–81. 10.5650/jos.62.873 24200934

[33] MaAG GeS ZhangM ShiXX SchoutenEG KokFJ . Antioxidant micronutrients improve intrinsic and UV-induced apoptosis of human lymphocytes particularly in elderly people. J Nutr Health Aging. (2011) 15:912–7. 10.1007/s12603-011-0118-122159782

[34] Jacob K Periago MJ Böhm V Berruezo GR. Influence of lycopene and vitamin C from tomato juice on biomarkers of oxidative stress and inflammation. Br J Nutr. (2008) 99:137–46. 10.1017/S0007114507791894 17640421

[35] Briviba K Bub A Möseneder J Schwerdtle T Hartwig A Kulling S . No differences in DNA damage and antioxidant capacity between intervention groups of healthy, nonsmoking men receiving 2, 5, or 8 servings/day of vegetables and fruit. Nutr Cancer. (2008) 60:164–70. 10.1080/01635580701621346 18444147

[36] Concentrate J Nantz MP Rowe C Percival SS. Immunity and antioxidant capacity in humans is enhanced by consumption of a dried, encapsulated fruit and vegetable juice concentrate. J Nutr. (2006) 136:2606–10. 10.1093/jn/136.10.2606 16988134

[37] Tauler P Aguilo A Gimeno I Fuentespina E Tur JA Pons A. Response of blood cell antioxidant enzyme defences to antioxidant diet supplementation and to intense exercise. Eur J Nutr. (2005) 45:187–95. 10.1007/s00394-005-0582-7 16365696

[38] Aust O Stahl W Sies H Tronnier H Heinrich U. Supplementation with tomato-based products increases lycopene, phytofluene, and phytoene levels in human serum and protects against UV-light-induced erythema. Int J Vitam Nutr Res. (2005) 75:54–60. 10.1024/0300-9831.75.1.54 15830922

[39] Hininger I Favier M Arnaud J Thoulon JM Hariveau E Favier A . Effects of a combined micronutrient supplementation on maternal biological status and newborn anthropometrics measurements: a randomized double-blind, placebo-controlled trial in apparently healthy pregnant women. Eur J Clin Nutr. (2004) 58:52–9. 10.1038/sj.ejcn.1601745 14679367

[40] Upritchard JE Schuurman CR Wiersma A Tijburg LBM Coolen SAJ Rijken PJ . Spread supplemented with moderate doses of vitamin E and carotenoids reduces lipid peroxidation in healthy, nonsmoking adults. Am J Clin Nutr. (2003) 78:985–92. 10.1093/ajcn/78.5.985 14594786

[41] Nelson JL Bernstein PS Schmidt MC Tress MSV Askew EW. Dietary modification and moderate antioxidant supplementation differentially affect serum carotenoids, antioxidant levels and markers of oxidative stress in older humans. J Nutr. (2003) 133:117–3123. 10.1093/jn/133.10.3117 14519794

[42] Kiokias S Gordon MH. Dietary supplementation with a natural carotenoid mixture decreases oxidative stress. Eur J Clin Nutr. (2003) 57:1135–40. 10.1038/sj.ejcn.1601655 12947433

[43] Heinrich U Gärtner C Wiebusch M Eichler O Sies H Tronnier H . Supplementation with beta-carotene or a similar amount of mixed carotenoids protects humans from UV-induced erythema. J Nutr. (2003) 133:98–101. 10.1093/jn/133.1.98 12514275

[44] Schmidt MC Askew EW Roberts DE Prior RL Ensign WY Jr Hesslink RE Jr. Oxidative stress in humans training in a cold, moderate altitude environment and their response to a phytochemical antioxidant supplement. Wilderness Environ Med. (2002) 13:94–105. 10.1580/1080-6032(2002)013[0094:OSIHTI]2.0.CO;2 12092978

[45] Stahl W Heinrich U Wiseman S Eichler O Sies H Tronnier H. Dietary tomato paste protects against ultraviolet light-induced erythema in humans. J Nutrc. (2001) 131:1449–51. 10.1093/jn/131.5.1449 11340098

[46] Stahl W Heinrich U Jungmann H Sies H Tronnier H. Carotenoids and carotenoids plus vitamin E protect against ultraviolet light-induced erythema in humans. Am J Clin Nutr. (2000) 71:795–8. 10.1093/ajcn/71.3.795 10702175

[47] Burrows TL Williams R Rollo M Wood L Garg ML Jensen M . Plasma carotenoid levels as biomarkers of dietary carotenoid consumption: a systematic review of the validation studies. J Nutr. (2015) 2:15–24. 10.1016/j.jnim.2015.05.001

[48] Prior RL Wu X Schaich K. Standardized methods for the determination of antioxidant capacity and phenolics in foods and dietary supplements. J Agric Food Chem. (2005) 53:4290–302. 10.1021/jf0502698 15884874

[49] Pisoschi AM Pop A Cimpeanu C Predoi G. Antioxidant capacity determination in plants and plant-derived products: a review. Oxid Med Cell Longev. (2016) 2016:913–76. 10.1155/2016/9130976 28044094 PMC5164913

[50] Shanely RA Nieman DC Perkins-Veazie P Henson DA Meaney MP Knab AM . Comparison of watermelon and carbohydrate beverage on exercise-induced alterations in systemic inflammation, immune dysfunction, and plasma antioxidant capacity. Nutrients. (2016) 8:518. 10.3390/nu8080518 27556488 PMC4997430

[51] Pellegrini N Serafini M Colombi B Del Rio D Salvatore S Bianchi M . Total antioxidant capacity of plant foods, beverages and oils consumed in Italy assessed by three different in vitro assays. J Nutr. (2003) 133:2812–9. 10.1093/jn/133.9.2812 12949370

[52] He L Eslamfam S Ma X Li D. Autophagy and the nutritional signaling pathway. Front Agr Sci Eng. (2016) 3:222–30. 10.15302/J-FASE-2016106

[53] He L He T Farrar S Ji L Liu T Ma X. Antioxidants maintain cellular redox homeostasis by elimination of reactive oxygen species. Cell Physiol Biochem. (2017) 44:532–53. 10.1159/000485089 29145191

[54] Lubos E Loscalzo J Handy DE. Glutathione peroxidase-1 in health and disease: from molecular mechanisms to therapeutic opportunities. Antioxid Redox Signal. (2011) 15:1957–97. 10.1089/ars.2010.3586 21087145 PMC3159114

[55] Franco R Navarro G Martínez-Pinilla E. Hormetic and mitochondria-related mechanisms of antioxidant action of phytochemicals. Antioxidants. (2019) 8:373. 10.3390/antiox8090373 31487950 PMC6769633

[56] Cornelius C Koverech G Crupi R Paola RD Koverech A Lodato F . Osteoporosis and alzheimer pathology: role of cellular stress response and hormetic redox signaling in aging and bone remodeling. Front Pharmacol. (2014) 5:120. 10.3389/fphar.2014.00120 24959146 PMC4050335

[57] Pan L Segrest JP. Computational studies of plasma lipoprotein lipids. Biochim Biophys Acta Biomembr. (2016) 1858:2401–20. 10.1016/j.bbamem.2016.03.010 26969087

[58] Ahotupa M: Oxidized lipoprotein lipids and atherosclerosis. Free Radic Res. (2017) 51:439–47. 10.1080/10715762.2017.1319944 28412863

[59] Li X Wei X Sun Y. High-fat diet promotes experimental colitis by inducing oxidative stress in the colon. Am J Physiol Gastrointest Liver Physiol. (2019) 317:453–62. 10.1152/ajpgi.00103.2019 31411504

[60] Lupachyk S Watcho P Hasanova N. Triglyceride, nonesterified fatty acids, and prediabetic neuropathy: role for oxidative-nitrosative stress. Free Radic Biol Med. (2012) 52:1255–63. 10.1016/j.freeradbiomed.2012.01.029 22366714 PMC3312982

[61] Chacón-Ordóñez T Carle R Schweiggert R. Bioaccessibility of carotenoids from plant and animal foods. J Sci Food Agric. (2019) 99:3220–39. 10.1002/jsfa.9525 30536912

[62] Failla ML Chitchumroonchokchai C Ishida BK. *In vitro* micellarization and intestinal cell uptake of cis isomers of lycopene exceed those of all-trans lycopene. J Nutr. (2008) 138:482–6. 10.1093/jn/138.3.482 18287353

[63] Burri B Neidlinger T Clifford AJ. Serum carotenoid depletion follows first-order kinetics in healthy adult women fed naturally low carotenoid diets. Nutr. (2001) 131:2096–100. 10.1093/jn/131.8.2096 11481400

